# 9-Ethyl-3,6-bis­(1*H*-imidazol-1-yl)-9*H*-carbazole

**DOI:** 10.1107/S1600536808013937

**Published:** 2008-05-14

**Authors:** Hong Ping Zhou, Liang Fei Lv, Peng Wang, Ren Tao Hu

**Affiliations:** a Department of Chemistry, Anhui University, Hefei 230039, Peoples Republic of China and, Key Laboratory of Opto-Electronic Information Acquisition and, Manipulation (Anhui University), Ministry of Education, Hefei 230039, People’s Republic of China

## Abstract

In the crystal structure of the title compound, C_20_H_17_N_5_, the two imidazole rings are twisted with respect to the carbazole plane, making dihedral angles of 55.8 (2) and 43.7 (2)°. The crystal structure is stabilized by weak C—H⋯N and C—H⋯π inter­actions.

## Related literature

For general background, see: Mi *et al.* (2003 ▶).
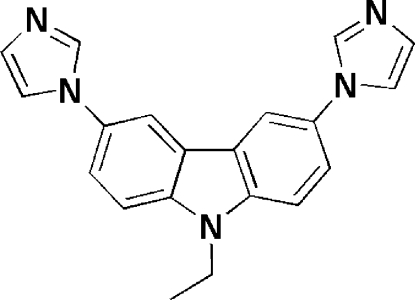


## Experimental

### 

#### Crystal data

C_20_H_17_N_5_
*M* *_r_* = 327.39
Triclinic, 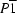

*a* = 5.625 (2) Å
*b* = 8.826 (3) Å
*c* = 17.367 (6) Å
α = 92.698 (6)°
β = 96.011 (6)°
γ = 102.567 (6)°
*V* = 834.8 (5) Å^3^
*Z* = 2
Mo *K*α radiation
μ = 0.08 mm^−1^
*T* = 293 (2) K
0.46 × 0.40 × 0.16 mm

#### Data collection

Bruker SMART APEX area-dectector diffractometer
Absorption correction: none
6040 measured reflections
2928 independent reflections
2611 reflections with *I* > 2σ(*I*)
*R*_int_ = 0.017

#### Refinement

*R*[*F* ^2^ > 2σ(*F* ^2^)] = 0.039
*wR*(*F* ^2^) = 0.120
*S* = 1.06
2928 reflections
225 parameters
H-atom parameters constrained
Δρ_max_ = 0.23 e Å^−3^
Δρ_min_ = −0.18 e Å^−3^

### 

Data collection: *SMART* (Bruker, 2002 ▶); cell refinement: *SAINT* (Bruker, 2002 ▶); data reduction: *SAINT*; program(s) used to solve structure: *SHELXS97* (Sheldrick, 2008 ▶); program(s) used to refine structure: *SHELXL97* (Sheldrick, 2008 ▶); molecular graphics: *ORTEPII* (Johnson, 1976 ▶); software used to prepare material for publication: *SHELXL97*.

## Supplementary Material

Crystal structure: contains datablocks I, global. DOI: 10.1107/S1600536808013937/xu2412sup1.cif

Structure factors: contains datablocks I. DOI: 10.1107/S1600536808013937/xu2412Isup2.hkl

Additional supplementary materials:  crystallographic information; 3D view; checkCIF report

## Figures and Tables

**Table 1:** Hydrogen-bond geometry (Å, °)

*D*—H⋯*A*	*D*—H	H⋯*A*	*D*⋯*A*	*D*—H⋯*A*
C1—H1⋯N1^i^	0.93	2.73	3.533 (2)	144
C2—H2⋯N1^ii^	0.93	2.63	3.452 (2)	148
C16—H16⋯N5^ii^	0.93	2.68	3.509 (2)	149
C14—H14⋯N5^iii^	0.93	2.66	3.570 (2)	165

Symmetry codes: (i) 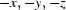; (ii) 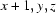; (iii) 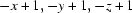.

**Fig. 1.**
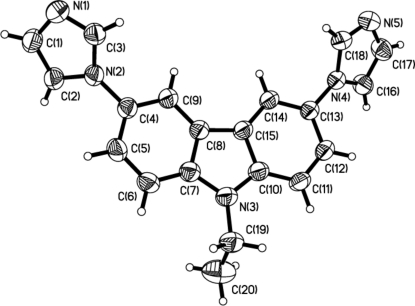
The molecular structure of the title compound showing 50% probability displacement ellipsoids.

## supplementary crystallographic information

### Comment

Carbazole derivatives exhibit good charge transfer and hole transporting properties, which are being explored for a multitude of optoelectronic and photocatalytic applications, including organic light emitting diodes (OLEDs) (Mi *et al.*, 2003). The title molecule containing imidazole with electrochemical and biology properties has been prepared, its crystal structure is reported here.

The molecular structure is shown in Fig. 1, the bond lengths and angles are normal. The dihedral angles between N2-imidazole and C4-benzene rings and between N4-imidazole and C10-benzene rings are 55.8 (2) and 43.7 (2)°, respectively. In the crystal structure, the molecules are stacked through the weak C19—H19A···*Cg*^i^ interactions (*Cg* is the centroid of the N1-imidazole ring), H19A···*Cg*^i^ = 2.85 Å, C19···*Cg*^i^ = 3.640 (11) Å and C19—H19A···*Cg*^i^ = 139° [symmetry code: (i) -1 + *x*, -1 + *y*, *z*]. The weak C—H···N hydrogen bonding (Table 1) helps to stabilize the crystal structure.

### Experimental

For the preparation of 3,6-diimidazolyl-9-ethylcarbazole, a mixture of CuI (0.27 g, 1.40 mmol) and 1,10-phenanthroline (0.60 g, 3.00 mmol) were heated at 393 K with DMF (3 ml) as solvent for 10 min. Then, the mixture was cooled to room temperature, potassium *tert*-butanol (6.05 g, 54.00 mmol), imidazole (3.65 g, 54.00 mmol), 3,6-diiodo-9-ethylcarbazole (3.00 g, 6.70 mmol) and 18-crown-6 (litter) were added and heated at 413 k for 48 h, then the reaction mixture was heated to 433 k for 12 h, and cooled to room temperature. The mixture solution was poured into water and extracted by dichloromethane. The organic layer was separated, dried with anhydrous magnesium sulfate. Then it was filtered and concentrated, the re-crystallization from ethyl acetate produced light yellow single crystals (1.50 g, Yield 70.0%).

### Refinement

All hydrogen atoms were placed in geometrically idealized positions and constrained to ride on their parent atoms, with C—H = 0.93 - 0.96 Å and *U*_iso_(H) = 1.2*U*_eq_(C) or 1.5*U*_eq_(C).

### Crystal data

**Table:**

C_20_H_17_N_5_	*Z* = 2
*M**_r_* = 327.39	*F*_000_ = 344
Triclinic, *P*1	*D*_x_ = 1.302 Mg m^−^^3^
Hall symbol: -P 1	Mo *K*α radiation λ = 0.71073 Å
*a* = 5.625 (2) Å	Cell parameters from 2928 reflections
*b* = 8.826 (3) Å	θ = 1.2–25.0º
*c* = 17.367 (6) Å	µ = 0.08 mm^−^^1^
α = 92.698 (6)º	*T* = 293 (2) K
β = 96.011 (6)º	Prism, yellow
γ = 102.567 (6)º	0.46 × 0.40 × 0.16 mm
*V* = 834.8 (5) Å^3^	

### Data collection

**Table:**

Bruker SMART APEX area-dectector diffractometer	2611 reflections with *I* > 2σ(*I*)
Radiation source: fine-focus sealed tube	*R*_int_ = 0.017
Monochromator: graphite	θ_max_ = 25.0º
*T* = 293(2) K	θ_min_ = 1.2º
φ and ω scans	*h* = −6→6
Absorption correction: none	*k* = −10→10
6040 measured reflections	*l* = −20→20
2928 independent reflections	

### Refinement

**Table:**

Refinement on *F*^2^	Hydrogen site location: inferred from neighbouring sites
Least-squares matrix: full	H-atom parameters constrained
*R*[*F*^2^ > 2σ(*F*^2^)] = 0.039	*w* = 1/[σ^2^(*F*_o_^2^) + (0.0695*P*)^2^ + 0.1471*P*] where *P* = (*F*_o_^2^ + 2*F*_c_^2^)/3
*wR*(*F*^2^) = 0.120	(Δ/σ)_max_ < 0.001
*S* = 1.06	Δρ_max_ = 0.23 e Å^−^^3^
2928 reflections	Δρ_min_ = −0.18 e Å^−^^3^
225 parameters	Extinction correction: SHELXL97 (Sheldrick, 2008), Fc^*^=kFc[1+0.001xFc^2^λ^3^/sin(2θ)]^-1/4^
Primary atom site location: structure-invariant direct methods	Extinction coefficient: 0.044 (6)
Secondary atom site location: difference Fourier map	

### Special details

**Table:**

Geometry. All e.s.d.'s (except the e.s.d. in the dihedral angle between two l.s. planes) are estimated using the full covariance matrix. The cell e.s.d.'s are taken into account individually in the estimation of e.s.d.'s in distances, angles and torsion angles; correlations between e.s.d.'s in cell parameters are only used when they are defined by crystal symmetry. An approximate (isotropic) treatment of cell e.s.d.'s is used for estimating e.s.d.'s involving l.s. planes.

### Fractional atomic coordinates and isotropic or equivalent isotropic displacement parameters (Å^2^)

**Table:**

	*x*	*y*	*z*	*U*_iso_*/*U*_eq_	
N2	0.49085 (19)	0.34017 (13)	0.10310 (6)	0.0435 (3)	
N4	0.89546 (19)	0.72250 (13)	0.53896 (6)	0.0418 (3)	
N5	0.6235 (2)	0.67394 (15)	0.62354 (7)	0.0553 (4)	
N1	0.1184 (2)	0.19639 (15)	0.06703 (7)	0.0535 (3)	
N3	1.2016 (2)	0.83688 (14)	0.24918 (7)	0.0484 (3)	
C13	0.9898 (2)	0.75062 (15)	0.46637 (7)	0.0405 (3)	
C5	0.8279 (3)	0.56084 (17)	0.09187 (8)	0.0485 (4)	
H5	0.8010	0.5396	0.0383	0.058*	
C9	0.7148 (2)	0.49327 (16)	0.21905 (7)	0.0432 (3)	
H9	0.6179	0.4289	0.2500	0.052*	
C12	1.1789 (2)	0.88178 (16)	0.46167 (8)	0.0459 (4)	
H12	1.2463	0.9456	0.5062	0.055*	
C7	1.0438 (2)	0.71535 (16)	0.20364 (8)	0.0438 (3)	
C10	1.1590 (2)	0.82136 (16)	0.32594 (8)	0.0433 (3)	
C11	1.2661 (2)	0.91729 (16)	0.39191 (8)	0.0476 (4)	
H11	1.3933	1.0032	0.3890	0.057*	
C8	0.8970 (2)	0.62019 (15)	0.25193 (7)	0.0416 (3)	
C14	0.8859 (2)	0.65280 (15)	0.40123 (7)	0.0430 (3)	
H14	0.7624	0.5652	0.4048	0.052*	
C2	0.5129 (3)	0.22459 (17)	0.05004 (8)	0.0493 (4)	
H2	0.6569	0.2093	0.0323	0.059*	
C18	0.6561 (2)	0.68650 (16)	0.55011 (8)	0.0480 (4)	
H18	0.5281	0.6722	0.5101	0.058*	
C15	0.9703 (2)	0.68841 (15)	0.33029 (7)	0.0416 (3)	
C1	0.2852 (3)	0.13828 (17)	0.02886 (8)	0.0515 (4)	
H1	0.2460	0.0517	−0.0065	0.062*	
C6	1.0107 (3)	0.68540 (17)	0.12343 (8)	0.0496 (4)	
H6	1.1090	0.7476	0.0920	0.060*	
C4	0.6822 (2)	0.46580 (16)	0.13922 (7)	0.0423 (3)	
C16	1.0248 (3)	0.73427 (18)	0.61152 (8)	0.0503 (4)	
H16	1.1944	0.7584	0.6235	0.060*	
C19	1.3664 (3)	0.96821 (17)	0.22027 (9)	0.0535 (4)	
H19A	1.3862	1.0591	0.2560	0.064*	
H19B	1.2921	0.9914	0.1705	0.064*	
C3	0.2492 (2)	0.31619 (17)	0.11053 (8)	0.0473 (4)	
H3	0.1832	0.3784	0.1432	0.057*	
C17	0.8562 (3)	0.70373 (19)	0.66187 (8)	0.0553 (4)	
H17	0.8929	0.7030	0.7153	0.066*	
C20	1.6114 (4)	0.9380 (3)	0.21097 (14)	0.0896 (6)	
H20A	1.6872	0.9167	0.2602	0.134*	
H20B	1.7120	1.0279	0.1923	0.134*	
H20C	1.5936	0.8501	0.1744	0.134*	

### Atomic displacement parameters (Å^2^)

**Table:**

	*U*^11^	*U*^22^	*U*^33^	*U*^12^	*U*^13^	*U*^23^
N2	0.0408 (6)	0.0507 (7)	0.0374 (6)	0.0095 (5)	0.0013 (4)	−0.0020 (5)
N4	0.0403 (6)	0.0420 (6)	0.0403 (6)	0.0042 (5)	0.0040 (5)	−0.0012 (5)
N5	0.0514 (7)	0.0583 (8)	0.0555 (8)	0.0067 (6)	0.0152 (6)	0.0044 (6)
N1	0.0427 (6)	0.0611 (8)	0.0533 (7)	0.0076 (6)	0.0006 (5)	0.0001 (6)
N3	0.0488 (7)	0.0466 (7)	0.0447 (7)	−0.0022 (5)	0.0084 (5)	0.0065 (5)
C13	0.0394 (7)	0.0409 (7)	0.0390 (7)	0.0047 (5)	0.0032 (5)	0.0012 (5)
C5	0.0521 (8)	0.0574 (9)	0.0353 (7)	0.0114 (7)	0.0047 (6)	0.0030 (6)
C9	0.0449 (7)	0.0436 (7)	0.0383 (7)	0.0042 (6)	0.0046 (5)	0.0022 (5)
C12	0.0446 (7)	0.0426 (8)	0.0448 (8)	0.0011 (6)	0.0003 (6)	−0.0031 (6)
C7	0.0438 (7)	0.0449 (8)	0.0416 (7)	0.0066 (6)	0.0056 (5)	0.0053 (6)
C10	0.0418 (7)	0.0415 (7)	0.0441 (7)	0.0032 (6)	0.0052 (5)	0.0050 (6)
C11	0.0429 (7)	0.0419 (8)	0.0508 (8)	−0.0048 (6)	0.0032 (6)	0.0029 (6)
C8	0.0433 (7)	0.0413 (7)	0.0380 (7)	0.0044 (6)	0.0051 (5)	0.0040 (5)
C14	0.0431 (7)	0.0388 (7)	0.0423 (7)	−0.0003 (6)	0.0040 (6)	0.0016 (6)
C2	0.0449 (8)	0.0552 (9)	0.0468 (8)	0.0118 (6)	0.0047 (6)	−0.0083 (6)
C18	0.0394 (7)	0.0499 (8)	0.0505 (8)	0.0019 (6)	0.0051 (6)	−0.0001 (6)
C15	0.0424 (7)	0.0381 (7)	0.0407 (7)	0.0015 (6)	0.0034 (5)	0.0035 (5)
C1	0.0513 (8)	0.0511 (8)	0.0483 (8)	0.0082 (7)	−0.0004 (6)	−0.0066 (6)
C6	0.0501 (8)	0.0552 (9)	0.0423 (8)	0.0051 (7)	0.0107 (6)	0.0101 (6)
C4	0.0397 (7)	0.0461 (8)	0.0400 (7)	0.0090 (6)	0.0017 (5)	−0.0001 (6)
C16	0.0441 (7)	0.0650 (9)	0.0411 (8)	0.0121 (6)	0.0021 (6)	0.0021 (6)
C19	0.0554 (9)	0.0474 (8)	0.0565 (9)	0.0047 (7)	0.0124 (7)	0.0122 (7)
C3	0.0406 (7)	0.0574 (9)	0.0442 (7)	0.0124 (6)	0.0045 (6)	0.0001 (6)
C17	0.0595 (9)	0.0679 (10)	0.0404 (8)	0.0165 (7)	0.0082 (6)	0.0074 (7)
C20	0.0618 (11)	0.0954 (15)	0.1158 (18)	0.0141 (10)	0.0283 (11)	0.0259 (13)

### Geometric parameters (Å, °)

**Table:**

N2—C3	1.3501 (17)	C7—C6	1.391 (2)
N2—C2	1.3769 (18)	C7—C8	1.4142 (18)
N2—C4	1.4336 (18)	C10—C11	1.3909 (19)
N4—C18	1.3512 (18)	C10—C15	1.4116 (19)
N4—C16	1.3750 (18)	C11—H11	0.9300
N4—C13	1.4299 (17)	C8—C15	1.4428 (19)
N5—C18	1.3135 (19)	C14—C15	1.3913 (19)
N5—C17	1.370 (2)	C14—H14	0.9300
N1—C3	1.3039 (18)	C2—C1	1.344 (2)
N1—C1	1.3748 (19)	C2—H2	0.9300
N3—C7	1.3867 (18)	C18—H18	0.9300
N3—C10	1.3867 (18)	C1—H1	0.9300
N3—C19	1.4629 (18)	C6—H6	0.9300
C13—C14	1.3844 (18)	C16—C17	1.350 (2)
C13—C12	1.403 (2)	C16—H16	0.9300
C5—C6	1.378 (2)	C19—C20	1.483 (2)
C5—C4	1.400 (2)	C19—H19A	0.9700
C5—H5	0.9300	C19—H19B	0.9700
C9—C4	1.3820 (19)	C3—H3	0.9300
C9—C8	1.3960 (18)	C17—H17	0.9300
C9—H9	0.9300	C20—H20A	0.9600
C12—C11	1.378 (2)	C20—H20B	0.9600
C12—H12	0.9300	C20—H20C	0.9600
			
C3—N2—C2	105.71 (11)	C1—C2—H2	126.8
C3—N2—C4	127.00 (11)	N2—C2—H2	126.8
C2—N2—C4	127.18 (11)	N5—C18—N4	112.78 (12)
C18—N4—C16	105.83 (11)	N5—C18—H18	123.6
C18—N4—C13	126.09 (11)	N4—C18—H18	123.6
C16—N4—C13	128.00 (11)	C14—C15—C10	119.99 (12)
C18—N5—C17	104.42 (12)	C14—C15—C8	133.50 (12)
C3—N1—C1	104.81 (11)	C10—C15—C8	106.43 (12)
C7—N3—C10	108.52 (11)	C2—C1—N1	110.27 (13)
C7—N3—C19	125.61 (12)	C2—C1—H1	124.9
C10—N3—C19	125.58 (12)	N1—C1—H1	124.9
C14—C13—C12	121.06 (12)	C5—C6—C7	118.08 (13)
C14—C13—N4	119.69 (12)	C5—C6—H6	121.0
C12—C13—N4	119.18 (11)	C7—C6—H6	121.0
C6—C5—C4	120.98 (13)	C9—C4—C5	121.53 (13)
C6—C5—H5	119.5	C9—C4—N2	119.89 (12)
C4—C5—H5	119.5	C5—C4—N2	118.56 (12)
C4—C9—C8	118.25 (13)	C17—C16—N4	106.22 (13)
C4—C9—H9	120.9	C17—C16—H16	126.9
C8—C9—H9	120.9	N4—C16—H16	126.9
C11—C12—C13	120.91 (12)	N3—C19—C20	112.98 (14)
C11—C12—H12	119.5	N3—C19—H19A	109.0
C13—C12—H12	119.5	C20—C19—H19A	109.0
N3—C7—C6	129.66 (13)	N3—C19—H19B	109.0
N3—C7—C8	108.93 (12)	C20—C19—H19B	109.0
C6—C7—C8	121.33 (13)	H19A—C19—H19B	107.8
N3—C10—C11	129.53 (13)	N1—C3—N2	112.77 (12)
N3—C10—C15	109.31 (12)	N1—C3—H3	123.6
C11—C10—C15	121.11 (13)	N2—C3—H3	123.6
C12—C11—C10	118.32 (13)	C16—C17—N5	110.75 (13)
C12—C11—H11	120.8	C16—C17—H17	124.6
C10—C11—H11	120.8	N5—C17—H17	124.6
C9—C8—C7	119.82 (12)	C19—C20—H20A	109.5
C9—C8—C15	133.27 (12)	C19—C20—H20B	109.5
C7—C8—C15	106.82 (12)	H20A—C20—H20B	109.5
C13—C14—C15	118.58 (12)	C19—C20—H20C	109.5
C13—C14—H14	120.7	H20A—C20—H20C	109.5
C15—C14—H14	120.7	H20B—C20—H20C	109.5
C1—C2—N2	106.44 (12)		
			
C18—N4—C13—C14	−44.43 (19)	C13—C14—C15—C8	−175.75 (14)
C16—N4—C13—C14	139.43 (14)	N3—C10—C15—C14	−176.74 (11)
C18—N4—C13—C12	132.48 (14)	C11—C10—C15—C14	1.0 (2)
C16—N4—C13—C12	−43.66 (19)	N3—C10—C15—C8	0.35 (15)
C14—C13—C12—C11	0.2 (2)	C11—C10—C15—C8	178.06 (12)
N4—C13—C12—C11	−176.63 (11)	C9—C8—C15—C14	−0.2 (3)
C10—N3—C7—C6	176.82 (13)	C7—C8—C15—C14	176.22 (14)
C19—N3—C7—C6	2.8 (2)	C9—C8—C15—C10	−176.67 (14)
C10—N3—C7—C8	0.07 (15)	C7—C8—C15—C10	−0.30 (15)
C19—N3—C7—C8	−173.96 (13)	N2—C2—C1—N1	0.16 (17)
C7—N3—C10—C11	−177.73 (13)	C3—N1—C1—C2	−0.17 (17)
C19—N3—C10—C11	−3.7 (2)	C4—C5—C6—C7	−0.9 (2)
C7—N3—C10—C15	−0.26 (16)	N3—C7—C6—C5	−175.61 (13)
C19—N3—C10—C15	173.77 (13)	C8—C7—C6—C5	0.8 (2)
C13—C12—C11—C10	1.1 (2)	C8—C9—C4—C5	0.6 (2)
N3—C10—C11—C12	175.49 (13)	C8—C9—C4—N2	−177.63 (11)
C15—C10—C11—C12	−1.7 (2)	C6—C5—C4—C9	0.3 (2)
C4—C9—C8—C7	−0.7 (2)	C6—C5—C4—N2	178.49 (12)
C4—C9—C8—C15	175.30 (14)	C3—N2—C4—C9	55.86 (19)
N3—C7—C8—C9	177.10 (12)	C2—N2—C4—C9	−128.49 (15)
C6—C7—C8—C9	0.0 (2)	C3—N2—C4—C5	−122.38 (15)
N3—C7—C8—C15	0.15 (15)	C2—N2—C4—C5	53.27 (19)
C6—C7—C8—C15	−176.93 (13)	C18—N4—C16—C17	0.40 (16)
C12—C13—C14—C15	−1.0 (2)	C13—N4—C16—C17	177.15 (13)
N4—C13—C14—C15	175.86 (11)	C7—N3—C19—C20	−89.67 (19)
C3—N2—C2—C1	−0.09 (16)	C10—N3—C19—C20	97.30 (19)
C4—N2—C2—C1	−176.48 (13)	C1—N1—C3—N2	0.11 (16)
C17—N5—C18—N4	0.02 (16)	C2—N2—C3—N1	−0.02 (16)
C16—N4—C18—N5	−0.26 (16)	C4—N2—C3—N1	176.39 (12)
C13—N4—C18—N5	−177.10 (12)	N4—C16—C17—N5	−0.41 (18)
C13—C14—C15—C10	0.4 (2)	C18—N5—C17—C16	0.24 (17)

### Hydrogen-bond geometry (Å, °)

**Table:**

*D*—H···*A*	*D*—H	H···*A*	*D*···*A*	*D*—H···*A*
C1—H1···N1^i^	0.93	2.73	3.533 (2)	144
C2—H2···N1^ii^	0.93	2.63	3.452 (2)	148
C16—H16···N5^ii^	0.93	2.68	3.509 (2)	149
C14—H14···N5^iii^	0.93	2.66	3.570 (2)	165

Symmetry codes: (i) −*x*, −*y*, −*z*; (ii) *x*+1, *y*, *z*; (iii) −*x*+1, −*y*+1, −*z*+1.
